# Supplementary material for: DiffuSETS: 12-Lead ECG generation conditioned on clinical text reports and patient-specific information
Source: Patterns (N Y). 2025 Jun 11;6(10):101291. doi: 10.1016/j.patter.2025.101291 (PMC12546759; doi:10.1016/j.patter.2025.101291)
Supplement: Document S1. Table S1 and Figures S1–S4 [file mmc1.pdf]

Patterns, Volume *6*

Supplemental information

DiffuSETS: 12-Lead ECG generation conditioned on clinical text reports and patient-specific information

Yongfan Lai, Jiabo Chen, Qinghao Zhao, Deyun Zhang, Yue Wang, Shijia Geng, Hongyan Li, and Shenda Hong

Table S1

Table S1. Extended Performance Comparison Between Baselines and DiffuSETS. At the signal level, we report the average amplitude of characteristic ECG waves at Lead-I, measured using the FeatureDB toolbox. The values in parentheses are the absolute error relative to the real signal (Hence, the lower is better). The best values are bolded, while the second-best values are underlined.

Source	Signal Level			Feature Level	Diagnostic Level
	Avg. P	Avg. QRS	Avg. T	Heart Rate MAE (↓)	CLIP (↑)
Real	0.022	0.894	0.039	-	-
WAVEGAN	0.049 (0.027)	0.835 (0.059)	0.048 (0.009)	40.52	0.648
Pulse2Pulse	**0.026 (0.004)**	0.824 (0.070)	**0.040 (0.001)**	40.63	<u>0.659</u>
SSSD-ECG	0.011 (0.011)	**0.922 (0.028)**	0.034 (0.005)	<u>37.81</u>	0.623
DiffuSETS	<u>0.013 (0.009)</u>	**0.866 (0.028)**	**0.040 (0.001)**	**6.73**	**0.795**

Figure S1

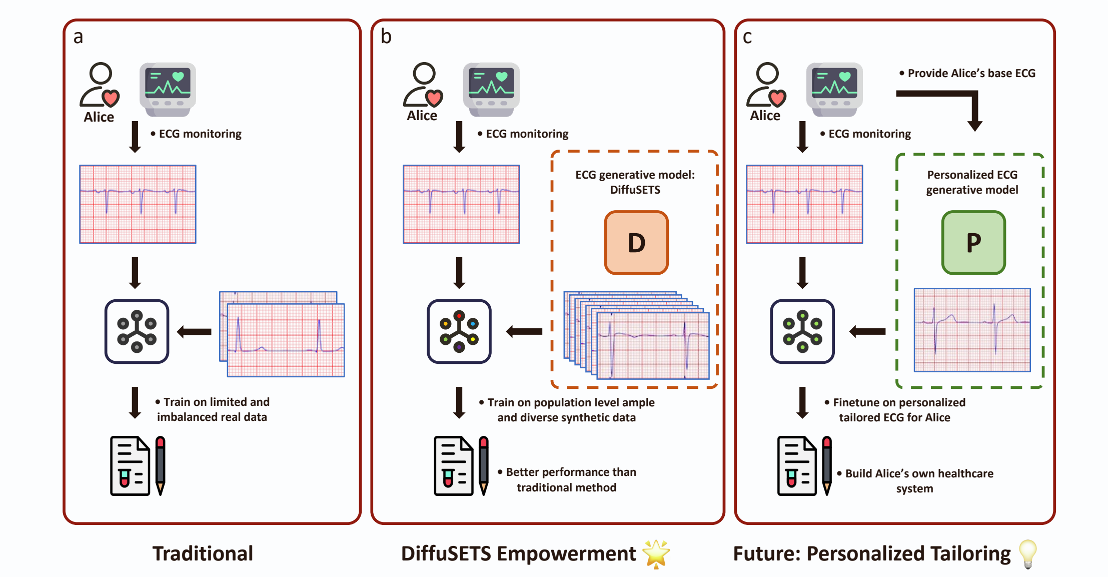

Figure S1. Clinical workflow of ECG auto diagnosis.

(a) Traditional Approach: Alice's real-time ECG is diagnosed by model trained on limited and imbalanced real data.

(b) DiffuSETS Empowerment (Ours): Alice's real-time ECG is diagnosed by model trained on ample of diverse dataset synthesized by DiffuSETS, leading to enhanced diagnostic performance.

(c) Personalized Tailoring (Future): Alice first provides her ECG to a personalized ECG generative model, which then generates digital twins of her ECG under various conditions (e.g., myocardial infarction). The ECG auto-diagnosis model is then finetuned on Alice's own ECG data, transforming it into a personalized ECG diagnosis model tailored specifically for her.

Figure S2

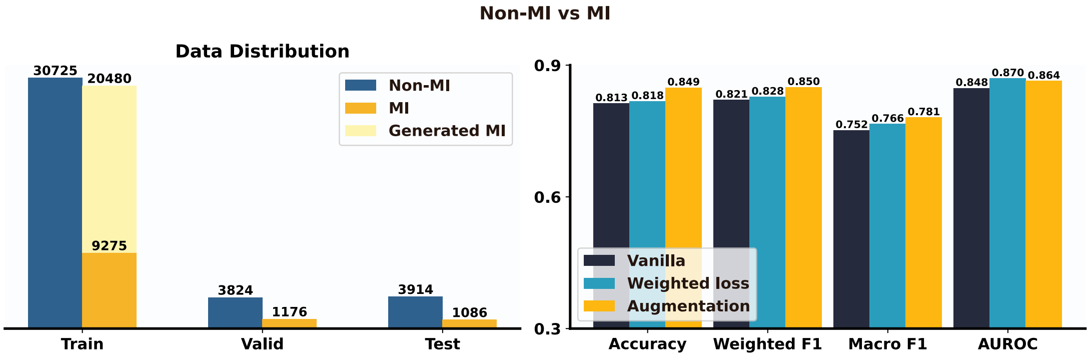

Figure S2. DiffuSETS-synthesized data can enhance the performance of myocardial infarction diagnosis.

Figure S3

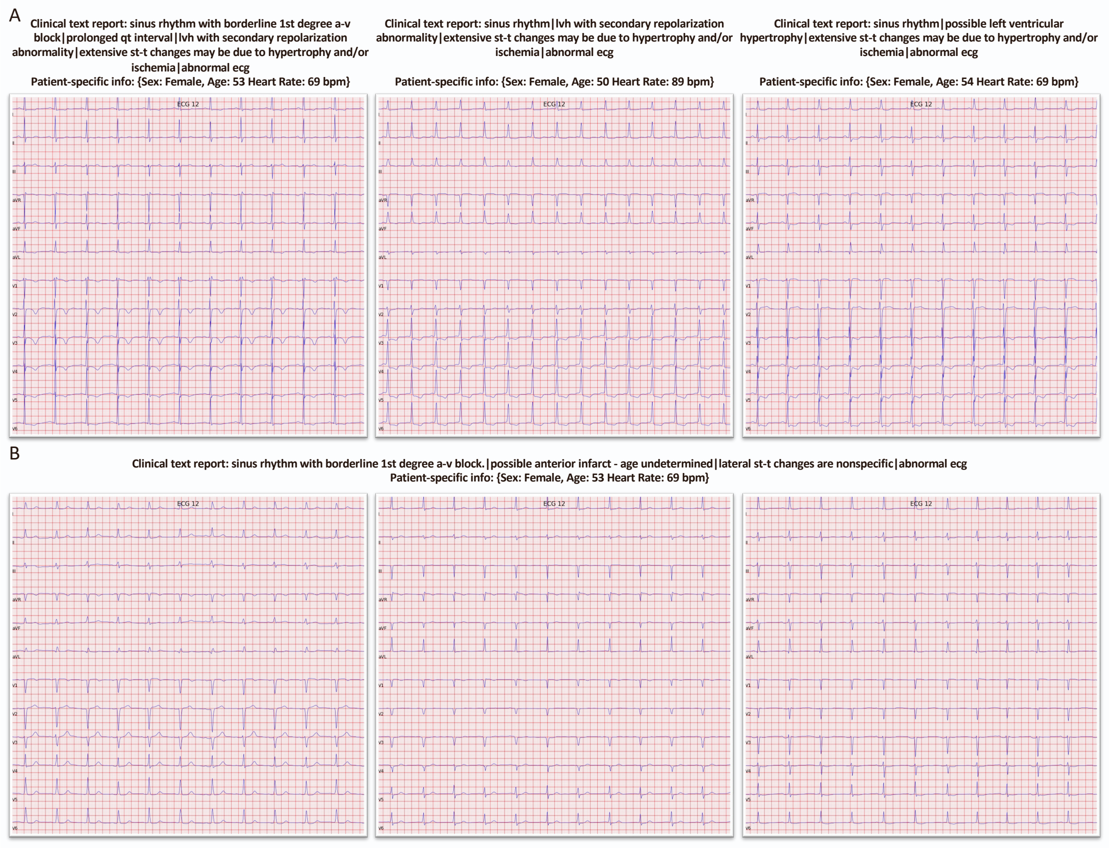

Figure S3. Case study of DiffuSETS outputs diverse ECGs.

(A) Myocardial ischemia ECGs based on different textual descriptions.

(B) Myocardial infarction ECGs from the same input.

Figure S4

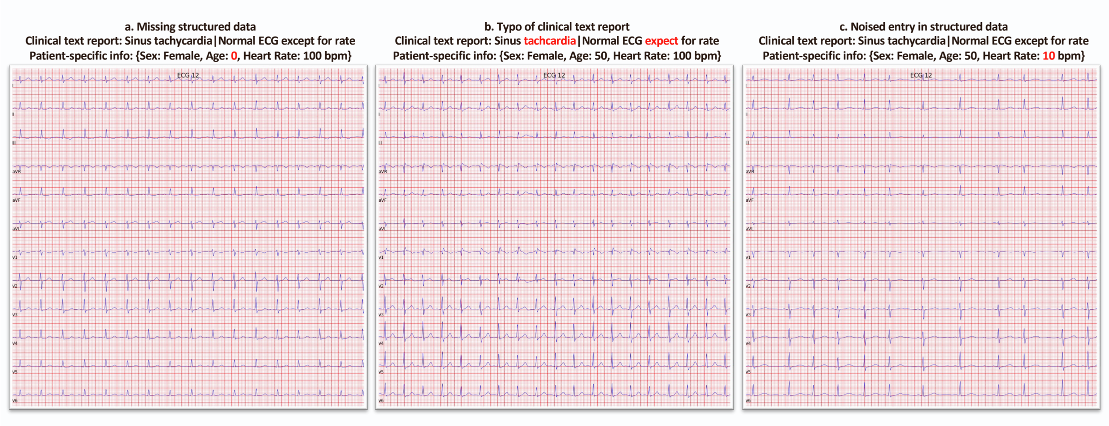

Figure S4. Case study of DiffuSETS outputs under noised input.

The original input is *Clinical text report: Sinus tachycardia|Normal ECG except for rate, Patient-specific info: {Sex: Female, Age: 52, Heart Rate: 100 bpm}*

(a) Missing structured data (age): The input age is set to zero. During generation, if detected as an outlier, the age is reset to the population average (i.e., 62).

(b) Typo in the clinical text report: Credited to the robustness embeddings of utilized large language model, DiffuSETS successfully capture the intended meaning.

(c) Noised entry in structured data: This scenario simulates incorrect structured data input (A zero is missed while typing the heart rate). DiffuSETS mitigates these errors by integrating information from both the heart rate and the clinical text report, where the latter indicates that the ECG should reflect a heart rate above the average level.
